# Differential Effect of Left vs. Right White Matter Hyperintensity Burden on Functional Decline: The Northern Manhattan Study

**DOI:** 10.3389/fnagi.2017.00305

**Published:** 2017-09-20

**Authors:** Mandip S. Dhamoon, Ying-Kuen Cheung, Ahmet Bagci, Noam Alperin, Ralph L. Sacco, Mitchell S. V. Elkind, Clinton B. Wright

**Affiliations:** ^1^Department of Neurology, Icahn School of Medicine at Mount Sinai New York, NY, United States; ^2^Department of Epidemiology, Mailman School of Public Health, Columbia University New York, NY, United States; ^3^Department of Biostatistics, Mailman School of Public Health, Columbia University New York, NY, United States; ^4^Evelyn F. McKnight Brain Institute, Miller School of Medicine, University of Miami Miami, FL, United States; ^5^Departments of Public Health Sciences and Human Genetics, Miller School of Medicine, University of Miami Miami, FL, United States; ^6^Department of Neurology, College of Physicians and Surgeons, Columbia University New York, NY, United States; ^7^National Institutes of Health Bethesda, MD, United States

**Keywords:** white matter hyperintensities, disability, trajectory, MRI and fMRI, subclinical ischemia

## Abstract

Asymmetry of brain dysfunction may disrupt brain network efficiency. We hypothesized that greater left-right white matter hyperintensity volume (WMHV) asymmetry was associated with functional trajectories.

**Methods:** In the Northern Manhattan Study, participants underwent brain MRI with axial T1, T2, and fluid attenuated inversion recovery sequences, with baseline interview and examination. Volumetric WMHV distribution across 14 brain regions was determined separately by combining bimodal image intensity distribution and atlas based methods. Participants had annual functional assessments with the Barthel index (BI, range 0–100) over a mean of 7.3 years. Generalized estimating equations (GEE) models estimated associations of regional WMHV and regional left-right asymmetry with baseline BI and change over time, adjusted for baseline medical risk factors, sociodemographics, and cognition, and stroke and myocardial infarction during follow-up.

**Results:** Among 1,195 participants, greater WMHV asymmetry in the parietal lobes (−8.46 BI points per unit greater WMHV on the right compared to left, 95% CI −3.07, −13.86) and temporal lobes (−2.48 BI points, 95% CI −1.04, −3.93) was associated with lower overall function. Greater WMHV asymmetry in the parietal lobes (−1.09 additional BI points per year per unit greater WMHV on the left compared to right, 95% CI −1.89, −0.28) was independently associated with accelerated functional decline.

**Conclusions:** In this large population-based study with long-term repeated measures of function, greater regional WMHV asymmetry was associated with lower function and functional decline. In addition to global WMHV, WHMV asymmetry may be an important predictor of long-term functional status.

## Introduction

Functional status is a patient-centered outcome that quantifies the burden of a disease and its impact on individuals' participation in society. Among the elderly, the trajectory of functional status is complex and influenced by several factors (Dhamoon et al., 2014). In particular, MRI measures of subclinical vascular disease are emerging as important predictors of outcomes (Dhamoon et al., 2017). White matter hyperintensities (WMH) in the brain are likely caused by traditional vascular risk factors (Hofer et al., 2015), and they have been associated with subsequent stroke [hazard ratio (HR) 1.86, 95% confidence interval (CI) 1.35–2.56; (Ntaios et al., 2015)] mortality [HR 1.64 (1.37, 1.97) for WMH grade 3 vs. 0 or 1; (Kuller et al., 2007)], cognitive impairment (drop of 2.4 vs. 0.9 points on the Modified Mini-Mental State Examination; Longstreth et al., 2002; Hachinski, 2008) and functional impairment (with 2–3-fold greater impairment with greater WMH; Pohjasvaara et al., 2007; Baune et al., 2009; Poggesi et al., 2011).

We previously showed that overall brain white matter hyperintensity volume (WMHV) was associated with accelerated long-term functional decline, independently of vascular events (Dhamoon et al., 2015). However, it was unclear whether asymmetry of WMHV burden in particular brain regions is more predictive of decline, independently of overall WMHV. In the normal brain, there is asymmetric localization of specific brain functions such as, language, but reliable structural asymmetries have not been found. With individual patients, neurologists are able to anticipate the impact of an asymmetric acute vascular brain lesion on functional status (Yaghi and Elkind, 2015), but the influence of asymmetrical subclinical white matter changes on functional status among community-dwelling healthy individuals is uncertain. Asymmetry of WMHV has not been previously examined as an independent predictor of functional status, but it has the potential to independently explain functional trajectories, as brain networks involved in functional tasks are bilaterally distributed and can be impaired not only by total lesion burden but also asymmetry of burden (Volz et al., 2017).

We analyzed repeated measures of functional status in a large population-based imaging study, and examined the differential effect on functional trajectories of left vs. right WMHV in different brain regions; we will refer to this as examining the effect of *asymmetry* on functional trajectories. We hypothesized that greater WMHV asymmetry in particular brain regions would be more predictive of functional decline, independently of baseline confounders, vascular events occurring during follow-up, and total brain WMHV.

## Methods

The Northern Manhattan Study (NOMAS) prospective cohort includes an MRI substudy (as previously described by Elkind et al., 2006) that began in 2003 and included 1,290 individuals: (1) aged ≥50 years, (2) without MRI contraindications, (3) without clinical stroke, and (4) able to provide signed informed consent. Imaging was performed on a 1.5 T MRI system (Philips Medical Systems, Best, Netherlands), and included axial T1, axial T2, and Fluid Attenuated Inversion Recovery (FLAIR) sequences.

**Figure 1:**
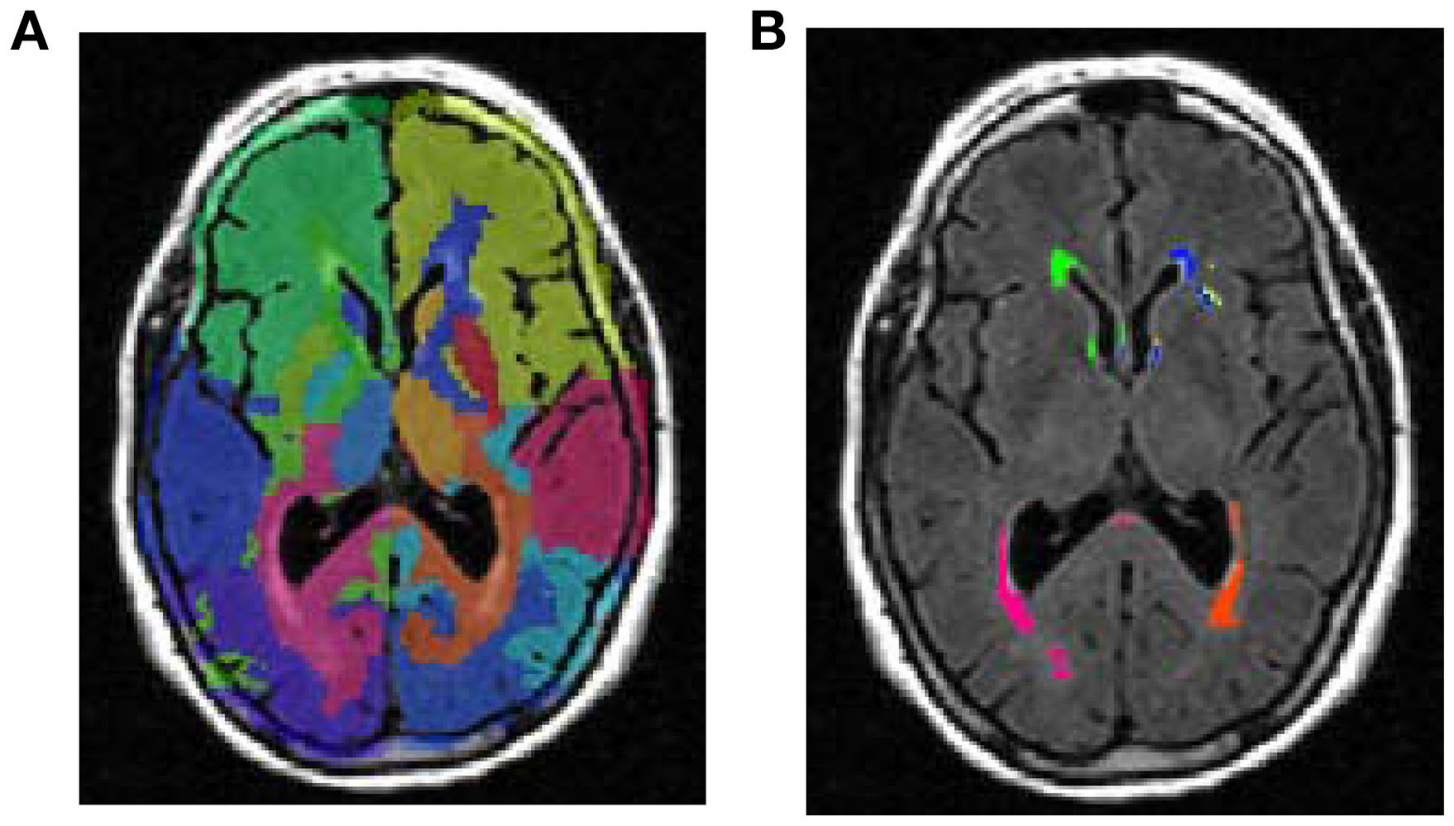
An example of lobar segmentation. **(A)** identification of brain lobes. **(B)** Measurement of white matter volumes in each identified lobe.

We developed tailored protocols using tools from the FSL software package (http://www.fmrib.ox.ac.uk/fsl) to automatically measure total WMHV and regional distributions over 14 brain regions [brainstem, cerebellum, and bilateral frontal, occipital, temporal, and parietal lobes, and bilateral anterior and posterior periventricular white matter (PVWM), defined as within 1 cm of the lateral ventricles; (DeCarli et al., 2005)]. The 14 regions selected for this study were chosen in an a priori fashion based upon standard neurological divisions into brain lobes. We did not use all available brain regions, greater than these 14, because the inclusion of too many regions reduces statistical power (by increasing the standard deviation) and reduces reliability especially in subjects who have an already low load of WMH volume. T2 FLAIR images were used for white matter segmentation. The skull region was removed from images using the FSL-Brain Extract Tool (Smith, 2002), and two-class segmentation of the whole brain was performed, into cerebrospinal fluid and brain tissue using the FSL-FAST segmentation algorithm after correction for image non-uniformity (Zhang et al., 2001). The mean (μ) and standard deviation (σ) of intensity values of the brain tissue voxels were derived, and WMH voxels were defined with intensity >(μ+3.5^*^σ). Finally, regional WMHV were calculated from T2-FLAIR derived images using the Montreal Neurological Institute structural atlas as a reference by nonlinearly registering the subject images to the atlas template and mapping the regions delineated on the atlas to the subject images using FSL-FNIRT (Non-Linear Image Registration) tool (Andersson et al., 2008). Total WMHV was the sum of volumes in each of the 14 sub-regions. The reliability of the WMH volumetry method was previously established using repeated measurements of the same subjects (Alperin et al., 2014). Total cranial volume (TCV) constituted the sum of whole brain volume voxels from the T1 segmentation process. Our segmentation methods differ from similar published methods (Iorio et al., 2013) because we calculated regional distribution, and optimized our thresholds for selection to minimize the need for manual editing. An example of segmentation images is provided in Figure 1.

Eighty-five participants were excluded due to: lack of FLAIR data, image artifacts in FLAIR data, including motion artifact, failure of the registration method due to very large ventricles or lesions/tumors, and lesions that appeared hyperintense outside the white matter. Columbia University and University of Miami IRBs approved the study and all participants provided written informed consent.

### Baseline evaluation

Bilingual research assistants collected interview data using standardized questions regarding the following conditions, as previously described: hypertension, diabetes mellitus, hypercholesterolemia, cigarette smoking, alcohol use, and cardiac conditions (Gentry et al., 1985). All participants underwent a thorough baseline examination including comprehensive medical history, physical examination, review of medical records, functional status assessed by the Barthel index (BI), and fasting blood samples.

### Follow-up

Participants were followed annually via phone to detect change in vital status, capture new neurological or cardiac symptoms and events and interval hospitalizations, and measure functional status via the BI. Only two subjects were lost to follow-up after their baseline examination, and the average annual contact rate was 99%. Participants were actively followed until this analysis.

A positive screen for any potential cardiac or neurological event was followed by an in-person assessment to determine whether a vascular outcome occurred. In addition, all admissions and discharges of NOMAS study participants to Columbia University Medical Center (CUMC) were screened for possible outcome events that may not have been captured by telephone interview. Hospital records were reviewed to classify outcomes as previously reported (Sacco et al., 2004). Stroke included ischemic stroke, intracerebral hemorrhage, and subarachnoid hemorrhage. At least two stroke neurologists verified and classified all stroke cases. MI required ≥2 of the three following criteria: (a) ischemic cardiac pain determined to be typical angina; (b) cardiac marker abnormalities defined as abnormal CK-MB fraction or troponin I values; and (c) ischemic EKG abnormalities. Cardiologists adjudicated diagnosis of MI independently after clinical data review. Through the end of 2014, there were 53 first definite and probable MI occurring during follow-up, and 64 first strokes (59 infarcts, 3 intracerebral hemorrhages, and 2 subarachnoid hemorrhages).

### Study outcome

The BI (Mahoney and Barthel, 1965; Granger et al., 1979) measures 10 activities of daily living (ADLs) and ranges from 0 to 100 in 5-point increments, with 100 indicating normal functioning. Previous research has demonstrated the reliability of phone assessments of function using the BI (Shinar et al., 1987). Although it is an ordinal scale, the scale may be analyzed as a continuous variable for increased power to detect associations, ability to describe the course of change over time in linear form, and avoidance of potential misclassification due to crude categorization (Song et al., 2006; Bath et al., 2007; Saver, 2011).

### Covariates

Analytic models were adjusted for the following variables: demographic variables (age, sex, race-ethnicity), medical risk factors [body mass index (body weight in kilograms divided by the square of height in meters), hypercholesterolemia (defined by self-report, lipid lowering therapy use, or fasting total cholesterol level >240 mg/dL), diabetes mellitus (defined by self-report, fasting blood glucose level ≥126 mg/dL, or insulin/oral hypoglycemic use), hypertension (defined as a systolic blood pressure recording ≥140 mmHg or a diastolic blood pressure recording ≥90 mm Hg based on the average of two blood pressure measurements or the participant's self-report of a history of hypertension or antihypertensive use)], smoking (defined as either non-smoker or smoker within the last year), alcohol use (with moderate alcohol use classified as 1 drink/month to 2 drinks/day), any physical activity (vs. none), social variables [marital status, insurance status (classified uninsured/Medicaid vs. Medicare/private insurance), and number of friends (individuals whom the participant knows well enough to visit in their homes)], and cognitive/mood factors [depressed mood and performance on mini-mental state examination (analyzed as a continuous variable)].

### Statistical analysis

We calculated distributions of regional WMHV, baseline covariates, and BI. All WMHV measures were calculated as percent of TCV. We calculated the frequency of direction of asymmetry (left > right vs. right > left) and percent right-left difference in each region [e.g., (right WMHV – left WMHV)/right WMHV].

**Figure 2:**
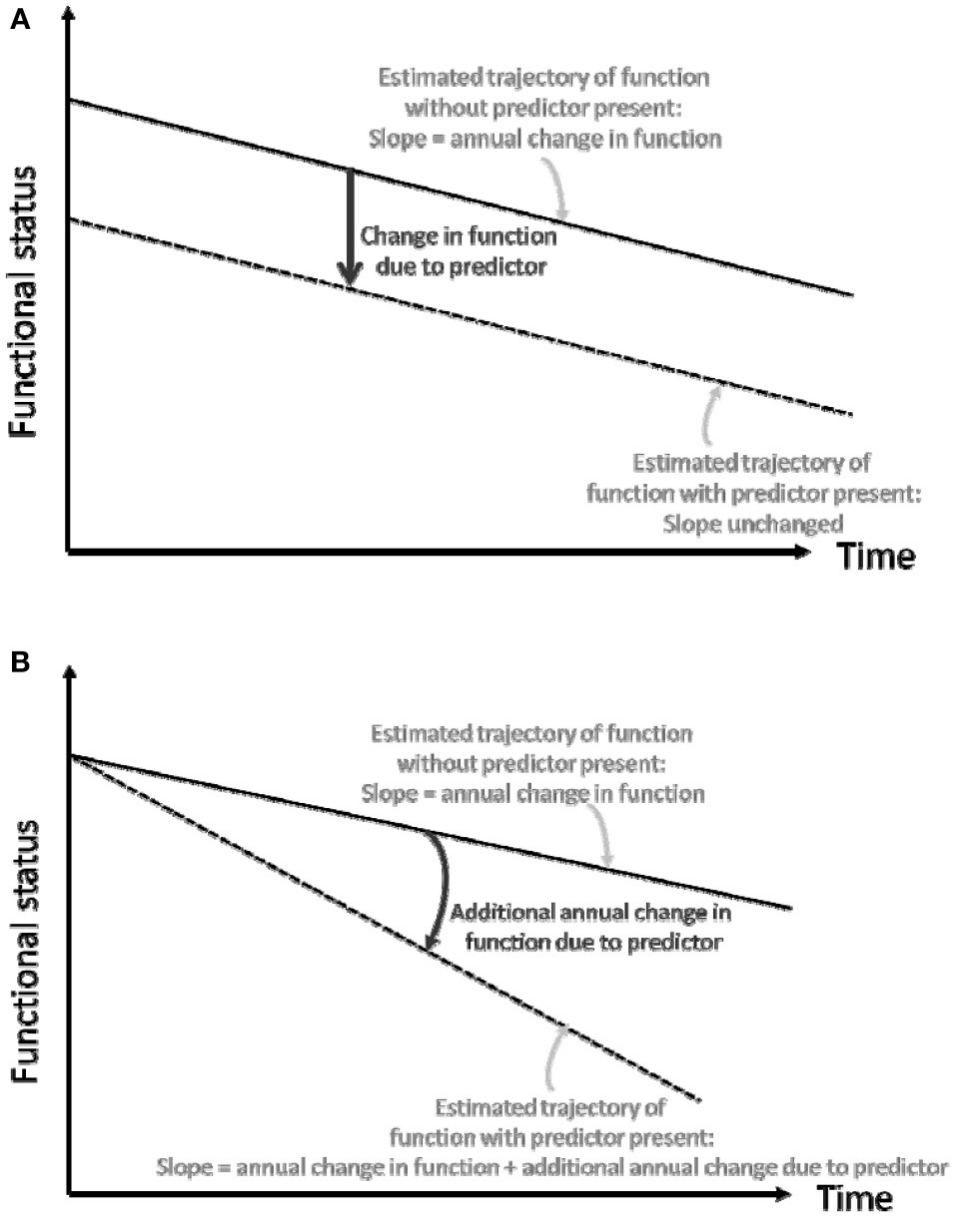
**(A)** Conceptual depiction of change in baseline functional status. **(B)** Conceptual depiction of change in slope of functional trajectory.

Next, we sought to determine whether regional WMHV asymmetry was associated with baseline BI (Figure 2A) and a steeper slope of decline over time (Figure 2B). Since a change in the baseline or intercept of the estimated curve shifts the entire curve, such a change can be considered a change in overall or mean function, but we will refer to this kind of change as change in baseline function. In order to estimate the differential effect of left vs. right WMHV in each region (which we refer to as “asymmetry”), two independent variables were included in models: a standardized left-sided WMHV measurement (“X1”), and the standardized left-sided WMHV + standardized right-sided WMHV (“X2”). The coefficient of X1 is equal to: (the average units increase in the outcome due to one SD increase in left-sided WMHV)—(the average units increase in the outcome due to one SD increase in right-sided WMHV). Thus, it measures the differential effects between the left and right WMHV on the outcome. If the coefficient is positive, then asymmetric WMHV burden (left > right) is associated with higher values of the outcome. In order to facilitate readers' interpretation of model estimates, we present the data graphically, with the point estimate of X1 and error bars for each region centered on a zero line. If greater WMHV burden on the left is associated with lower function, the graphed point will appear to the left of the zero line, and if greater WMHV burden on the right is associated with lower function, the graphed point will appear to the right of the zero line.

Due to correlations among repeated measures of BI in the same individual, regression models using generalized estimating equations (GEE) with an identity link function were used to assess the association between the above independent variables and repeated measurements of BI, in unadjusted models and adjusted for: baseline demographic variables, medical risk factors, smoking, and alcohol use and physical activity, social variables, and cognitive/mood factors, as defined above. In the fully adjusted models, we removed covariates not significant at a *p* = 0.2. In addition, in order to assess whether interval vascular events such as, clinical stroke and MI were implicated in the trajectory of functional status, we adjusted for stroke and MI as time-varying covariates. We also adjusted for total brain WMHV to assess whether the estimates of right-left asymmetry were independent of total brain WMHV.

In order to assess whether the independent variables (discussed above) were associated with change in BI over time (slope, Figure 2B), we included interaction terms between time of follow-up assessment and these variables. Various model diagnostics including tests of linearity, residual plots, and goodness of fit measures were used to evaluate the final model. There was no evidence to suggest lack of linearity of BI trajectories in the final models. As a working correlation structure for the GEE models we chose the exchangeable (intraclass) structure and compared the QIC obtained with this model with one using the unstructured working correlation structure.

We also examined whether the relationship between independent variables and functional trajectories differed for mobility (transfers, mobility, and stair use; maximum possible score 40) and non-mobility (feeding, bathing, grooming, dressing, bowels, bladder, and toilet use; maximum possible score 60) domains of the BI, which we analyzed separately in unadjusted and fully adjusted models as above. For tests of right-left asymmetry, we used a Bonferroni correction for the six brain regions with right and left sided measurements, so the *p*-value for significance for these tests was 0.05/6 = 0.008.

## Results

Table 1 shows distributions of baseline variables. Mean follow-up time, from MRI to last follow-up assessment, was 7.3 years (SD 2.1). Of 1,136 participants with BI of 95 or 100 at baseline and >1 follow-up, 690 (61.7%) experienced a decline in BI. Mean WMHV (as % TCV) by region are reported in Table 1, and the majority of WMHV burden involved the anterior and posterior periventricular regions. When the proportional right-left difference of regional WMHV was examined (Table 2), more individuals had right > left predominance for frontal, occipital, and parietal lobes and anterior and posterior periventricular regions, with the greatest proportional difference in the occipital lobe, followed by parietal and temporal lobes.

**Table 1:** Baseline characteristics of the cohort.

**Characteristic**	**Frequency**	
Number of participants, No. (%)	1290 (36.9)	
Age, mean (SD), y	64.5 (8.4)	
Body mass index, mean (SD), kg/m^2^	28.0 (4.8)	
Male, No. (%)	510 (39.5)	
Race-ethnicity:		
Non-Hispanic white, No. (%)	191 (14.8)	
Non-Hispanic black, No. (%)	223 (17.3)	
Hispanic, No. (%)	847 (65.7)	
Other, No. (%)	29 (2.3)	
Received at least high school education, No. (%)	592 (45.9)	
Marital status, No. (%) married	543 (42.1)	
Health insurance, NO. (%)		
Medicaid or no insurance	613 (47.5)	
Medicare or private insurance	677 (52.5)	
Hypertension	861 (66.7)	
Alcohol consumption:		
Never drank	264 (20.5)	
Past drinker	256 (19.8)	
Light drinker	163 (12.6)	
Moderate drinker	530 (41.1)	
Intermediate drinker	49 (3.8)	
Heavy drinker	28 (2.2)	
Physical activity		
None	564 (44.3)	
Any	710 (55.7)	
Diabetes mellitus:		
Smoking:		
Never	245 (19.0)	
Former	612 (47.4)	
Current	496 (38.5)	
Hypercholesterolemia	797 (61.8)	
History of coronary heart disease	177 (13.7)	
Hamilton depression scale score, mean (SD)	3.1 (3.8)	
Mini mental state score, mean (SD)	26.7 (3.3)	
Number of people known well enough to visit with		
in their homes:		
None	36 (2.8)	
1 or 2	124 (9.6)	
3 or 4	263 (20.4)	
5 or more	867 (67.2)	
Regional white matter hyperintensity volume, as % of total cranial volume, mean (SD)	**Left**	**Right**
Frontal lobe	0.051 (0.064)	0.058 (0.069)
Occipital lobe	0.005 (0.006)	0.005 (0.008)
Parietal lobe	0.018 (0.026)	0.023 (0.031)
Temporal lobe	0.017 (0.014)	0.017 (0.028)
Anterior periventricular	0.112 (0.154)	0.109 (0.150)
Posterior periventricular	0.150 (0.210)	0.154 (0.205)

**Table 2:** Summary statistics of right-left differences in white matter hyperintensity volumes^*^.

	**Predominance of white matter hyperintensity volume**				
	**Left > Right**		**Right > Left**		**Equal**
**Brain region**	***N***	**Percent difference, mean (*SD*)**	***N***	**Percent difference, mean (*SD*)**	***N***
Frontal lobe	493	20.7 (14.9)	668	29.1 (19.8)	34
Occipital lobe	450	51.8 (21.0)	557	53.6 (21.5)	188
Parietal lobe	357	29.8 (17.2)	762	38.3 (19.7)	76
Temporal lobe	655	29.6 (17.2)	471	30.1 (20.1)	69
Anterior periventricular	529	24.4 (17.5)	641	25.1 (17.6)	25
Posterior periventricular	466	20.6 (14.8)	718	24.5 (16.5)	11

* *Results are reported as a proportional difference in right-left asymmetry [e.g., (right WMHV–left WMHV)/right WMHV], and grouped into three categories: where left WMHV is greater than right WMHV; where right WMHV is greater than left WMHV; and where left and right WMHV was equal*.

**Figure 3:**
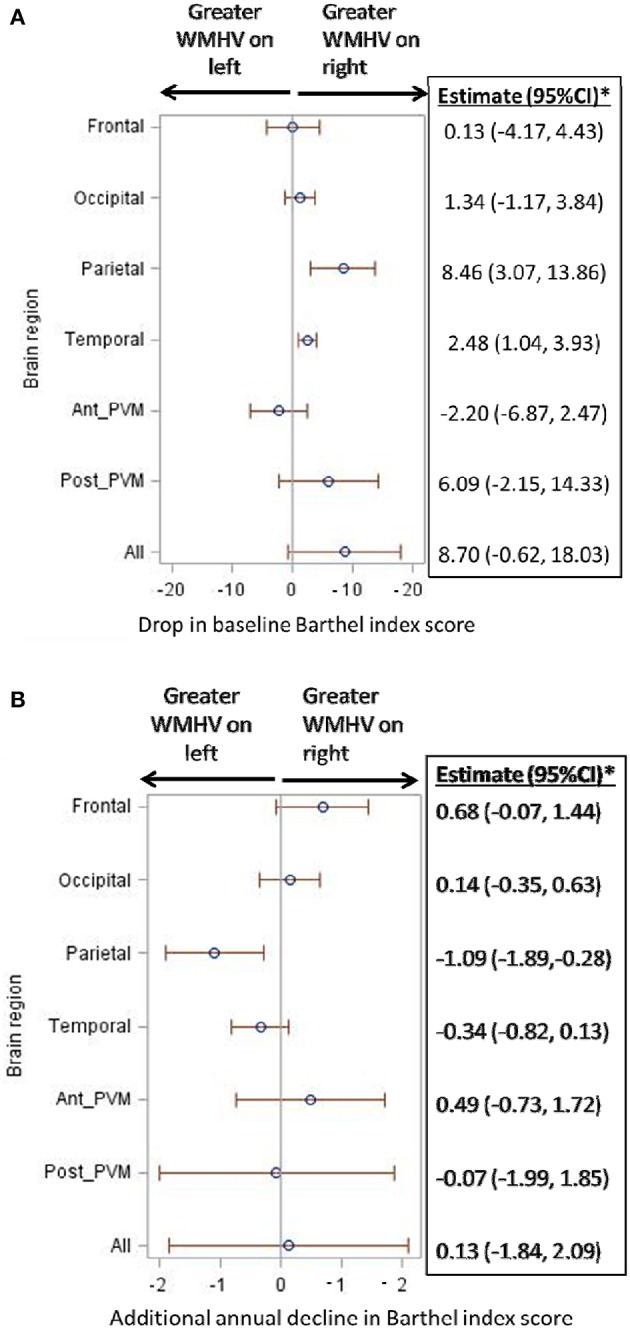
**(A)** Association of regional white matter hyperintensity volume asymmetry with baseline functional status. WMHV, white matter hyperintensity volume; Ant_PVM, anterior periventricular; Post_PVM, posterior periventricular. ^*^Estimates in the table are in reference to right-sided WMHV: a positive estimate signifies lower baseline Barthel index scores with greater WMHV on the right compared to the left. Models are adjusted for: age, sex, diabetes, hypertension, coronary artery disease, physical activity, alcohol use, body mass index, marital status, insurance status, mini-mental state score, total white matter hyperintensity volume, and stroke and myocardial infarction occurring during follow-up. **(B)** Association of regional white matter hyperintensity volume asymmetry with additional annual functional decline. WMHV, white matter hyperintensity volume; Ant_PVM, anterior periventricular; Post_PVM, posterior periventricular. ^*^Estimates in the table are in reference to right-sided WMHV: a positive estimate signifies additional annual decline in Barthel index scores with greater WMHV on the right compared to the left. Models are adjusted for: age, sex, diabetes, hypertension, coronary artery disease, physical activity, alcohol use, body mass index, marital status, insurance status, mini-mental state score, total white matter hyperintensity volume, and stroke and myocardial infarction occurring during follow-up.

Figure 3 shows results from fully adjusted models testing the association between regional right-left WMHV asymmetry and (1) baseline functional status (Figure 3A, conceptually depicted in Figure 2A) and (2) additional annual change in functional status (Figure 3B, conceptually depicted in Figure 2B). Greater asymmetry, with greater WMHV on the right compared to the left, was associated with lower baseline functional status in the parietal (−8.46 BI points per unit WMHV difference, 95% CI −3.07, −13.86) and temporal lobes (−2.48, 95% CI −1.04, −3.93). There was a trend for an association between greater WMHV asymmetry (right > left) in the entire brain and lower baseline BI score that did not reach significance (−8.70, 95% CI 0.62, −18.03).

When the effect of WMHV asymmetry on slope of functional status (Figure 2B) was examined, the estimated decline in functional status ranged from −0.97 to −1.03 BI points per year in all models. Asymmetry in the parietal region was associated with steeper annual decline: one unit greater WMHV burden in the left parietal lobe compared to the right was associated with an additional decline of −1.09 BI points per year (95% CI −1.89, −0.28, Figure 3B).

**Figure 4:**
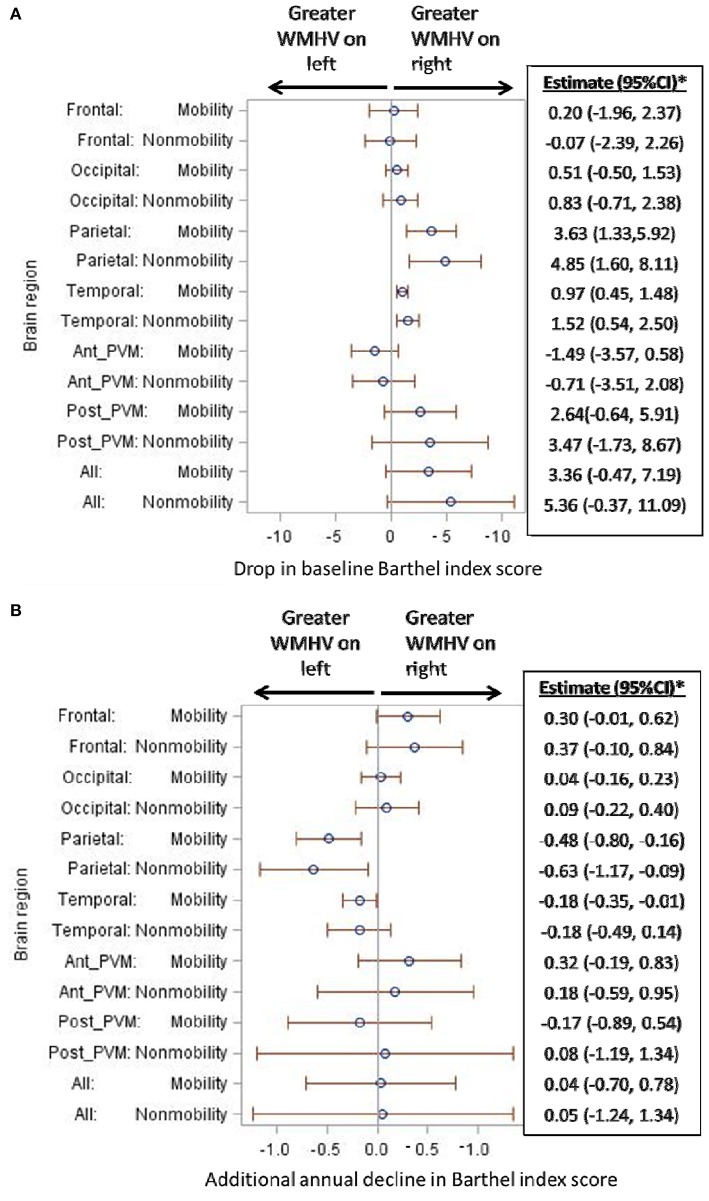
**(A)** Association of regional white matter hyperintensity volume asymmetry with baseline functional status, separately by mobility and non-mobility domains. WMHV, white matter hyperintensity volume; Ant_PVM, anterior periventricular; Post_PVM, posterior periventricular. ^*^Estimates in the table are in reference to right-sided WMHV: a positive estimate signifies lower baseline Barthel index scores with greater WMHV on the right compared to the left. Models are adjusted for: age, sex, diabetes, hypertension, coronary artery disease, physical activity, alcohol use, body mass index, marital status, insurance status, mini-mental state score, total white matter hyperintensity volume, and stroke and myocardial infarction occurring during follow-up. **(B)** Association of regional white matter hyperintensity volume asymmetry with additional annual functional decline, separately by mobility and non-mobility domains. WMHV, white matter hyperintensity volume; Ant_PVM, anterior periventricular; Post_PVM, posterior periventricular. ^*^Estimates in the table are in reference to right-sided WMHV: a positive estimate signifies additional annual decline in Barthel index scores with greater WMHV on the right compared to the left. Models are adjusted for: age, sex, diabetes, hypertension, coronary artery disease, physical activity, alcohol use, body mass index, marital status, insurance status, mini-mental state score, total white matter hyperintensity volume, and stroke and myocardial infarction occurring during follow-up.

When mobility and non-mobility domains of the BI were examined as separate outcomes (Figure 4), similar patterns as above persisted for each domain. Namely, there were associations of lower baseline BI score, in both mobility and non-mobility domains, with greater WMHV asymmetry (right > left) in the parietal and temporal lobes (Figure 4A). There was a trend for an association with WMHV asymmetry in the entire brain for each domain. When the slope of mobility and non-mobility domains of functional status was examined (Figure 4B), parietal WMHV asymmetry (left > right) was associated with steeper annual decline for both domains. In addition, asymmetry in the temporal lobe (left > right) was associated with steeper annual decline in the mobility domain: one unit greater WMHV burden on the left compared to the right was associated with an additional decline of −0.18 BI points per year (95% CI −0.35, −0.01).

## Discussion

We examined the differential effect, on functional trajectories, of left vs. right WMHV in different brain regions, which we term *asymmetry*. We found that greater asymmetry of WMHV in specific brain regions was associated with functional trajectories, independently of confounders, stroke and MI occurring during follow-up, and total brain WMHV. In particular, greater WMHV burden in the right parietal and temporal lobes, compared to the left, was associated with lower baseline functional status. Also, greater WMHV burden in the left parietal lobe, compared to the right, was associated with steeper annual functional decline, with a doubling of decline per unit right-left WMHV difference. Results were consistent when mobility and non-mobility functional domains were examined separately.

WMH in the brain are likely caused by ischemic processes (Conklin et al., 2014), but other presumed mechanisms include blood brain barrier disruption, inflammatory processes, ependymal loss, and venular dysfunction (Black et al., 2009). Rarer causes of white matter changes include cerebral amyloid angiopathy and Cerebral Autosomal Dominant Arteriopathy with Subcortical Infarcts and Leukoencephalopathy. The resulting WMH may disrupt cholinergic pathways involving the cortex, leading to dysfunction in executive, memory, and visuospatial domains that negatively impact functional status (Kim et al., 2011). However, these mechanisms do not explain why asymmetric WMHV may be associated with worse function. Although overall and regional WMHV have been tested as predictors of functional outcomes, the independent effect of WMHV asymmetry has been understudied.

Asymmetry is a fundamental aspect of normal brain function, with lateralization of language function, motor dominance, and perception (Toga and Thompson, 2003). However, in a healthy brain, macrostructural asymmetries have not been reliably found, either in gray or white matter volume, or sulcal or gyral sizes; the functional basis of the asymmetry may reside in lateralized synaptic networks or microstructural white matter tract differences (Büchel et al., 2004; de Schotten et al., 2011; Thiebaut de Schotten et al., 2011). It is conceivable that asymmetrical lesion burden or pathology in diverse neurological conditions may be associated with functional status, independently of total lesion burden. There are several possible justifications for such an association. Lateral injury may disrupt excitatory or inhibitory interhemispheric interactions that could impair performance of the complex tasks required for ADLs (Takeuchi et al., 2012). For example, asymmetry of frontal white matter fractional anisotropy measured by diffusion tensor imaging was associated with worse executive functioning (Yin et al., 2013). Among 11 individuals with recent ischemic stroke (Graziadio et al., 2012), the degree of asymmetry in corticospinal tract activity, measured by electroencephalography and electromyography, was independently associated with poorer recovery. It appears that symmetry of brain function and structure may be important for optimal functioning, and asymmetric WMHV burden is associated with worse functional trajectories, even when adjusting for total brain WMHV, as we have found. Furthermore, although asymmetry of vascular anatomy is common, especially in the transverse sinuses (Alper et al., 2004), the relationship between this asymmetry and white matter asymmetry has yet to be explored.

We found that greater WMHV burden in the right parietal and temporal lobes, compared to the left, was associated with lower baseline functional status. Right-sided clinical stroke has been previously associated with accelerated functional decline over the long term, independently of age, vascular risk factors, and stroke severity (Dhamoon et al., 2009). Subclinical WMHV may similarly cause impaired cross-sectional or baseline functional status. Also, greater WMHV burden in the left parietal lobe, compared to the right, was associated with steeper annual functional decline, with a doubling of decline per unit right-left WMHV difference. Disruption of pathways involved in parietal association cortices may impact the cognitive and visuospatial aspects of functional status and result in accelerated long-term decline. These patterns of association were consistent when mobility and non-mobility functional domains were examined separately, suggesting that asymmetric white matter injury not only affects motor pathways but also those that are involved in executive functioning, visuospatial perception, and ordering of complex tasks.

We previously showed, in this cohort, that periventricular WMHV was uniquely selected, among all tested brain regions, with accelerated functional decline over time (Dhamoon et al., 2017). Indeed, multiple studies have shown that periventricular WMHV may develop earlier, and with greater burdens, than in other brain regions (Wakefield et al., 2010; Moscufo et al., 2011; Zheng et al., 2012). Greater periventricular WMHV has been associated with lower mobility and gait speed (Moscufo et al., 2011), physical decline (Zheng et al., 2012), and falls (Blahak et al., 2009). However, we found no association between periventricular WMHV asymmetry and functional trajectories, likely because periventricular WMHV accumulates symmetrically over time (Gunning-Dixon et al., 2009). Indeed, the smallest differences in right-left WMHV in our study were seen in periventricular regions. Periventricular WMH have been associated with impaired insight and different temperament profiles were associated with differences in the subcortical structures of the brain (Serafini et al., 2016). Those with lower insight had significantly more periventricular WMH when compared to patients with higher insight. Also, differences among temperament groups were associated with differences in WMH patterns (Serafini et al., 2011). Further study would clarify relationships between WMH patterns and factors that affect performance in ADLs.

Strengths of this study include the large population-based cohort, accurate assessment of events during follow-up, minimal loss to follow-up, the use of state-of-the-art imaging and regional measurement of WMHV, and repeated measures of functional outcomes that allow trajectory analysis. As a result of these strengths, this is the first study to our knowledge to examine asymmetrical WMHV and associations with functional trajectories. A limitation of this study is that, in the MRI substudy, participants were recruited from the prospective cohort and most often obtained MRI imaging during follow-up instead of at baseline. The NOMAS participants enrolled in the MRI cohort were able to return for follow up and undergo MR imaging, reflecting a healthy survivor bias, which may have reduced power to detect declines in functional status. Also, although the ascertainment of follow-up events is excellent in NOMAS, there is the chance that some events occurring during follow-up may not have been captured. Finally, 1.5 T MRI was used in this study, and there is the chance that using 3 T MRI would have resulted in greater resolution for WMHV and greater overall volumes measured.

In conclusion, we found that greater asymmetry of WMHV in particular brain regions was independently associated with worse functional trajectories over time. These findings suggest that not only overall burden of WMHV, but also degree of asymmetry of this burden, may be important independent prognostic signs of functional limitations. Further research will clarify whether asymmetric WMHV causes impairment by disrupting interhemispheric brain networks, over and above direct damage to motor and association pathways.

## Ethics statement

This study was carried out in accordance with the recommendations of the IRB of Columbia University and the University of Miami with written informed consent from all subjects. All subjects gave written informed consent in accordance with the Declaration of Helsinki. The protocol was approved by the IRB of Columbia University and the University of Miami.

## Author contributions

MD: study design, interpretation, analysis, and writing. YC, AB, and NA: analysis and writing. RS: study design and writing. ME and CW: study design, analysis, and writing.

### Conflict of interest statement

The authors declare that the research was conducted in the absence of any commercial or financial relationships that could be construed as a potential conflict of interest.
